# Efficacy and Toxicity in Scheduled Intravesical Gemcitabine Versus Bacillus Calmette–Guérin for Bladder Cancer: A Systematic Review and Meta-Analysis

**DOI:** 10.3390/cancers18060990

**Published:** 2026-03-18

**Authors:** Do Kyung Kim, Jinhyung Jeon, Jong Kyou Kwon, Sungun Bang, Kwang Suk Lee, Kyo Chul Koo, Kang Su Cho

**Affiliations:** 1 Department of Urology, Prostate Cancer Center, Gangnam Severance Hospital, Urological Science Institute, College of Medicine, Yonsei University, Seoul 06273, Republic of Korea; dokyung80@yuhs.ac (D.K.K.); jkstorm@yuhs.ac (J.K.K.); bbsungun@yuhs.ac (S.B.); calmenow@yuhs.ac (K.S.L.); gckoo@yuhs.ac (K.C.K.); 2 Department of Urology, Yongin Severance Hospital, College of Medicine, Yonsei University, Yongin 16995, Republic of Korea; jun1644@yuhs.ac; 3 Center of Evidence Based Medicine, Institute of Convergence Science, Yonsei University, Seoul 03722, Republic of Korea

**Keywords:** gemcitabine, Bacillus Calmette–Guérin, intravesical, bladder cancer, meta-analysis

## Abstract

**Background/Objectives**: The present study aimed to compare the efficacy and safety of intravesical gemcitabine versus Bacillus Calmette–Guérin administered after transurethral resection of bladder tumor in patients with non-muscle-invasive bladder cancer. **Methods**: A comprehensive literature search was conducted using PubMed, Embase, and the Cochrane Library databases up to September 2025. Studies were eligible for inclusion if they were comparative studies evaluating intravesical gemcitabine versus Bacillus Calmette–Guérin following transurethral resection of bladder tumor. The primary outcomes were recurrence-free survival, progression-free survival, and incidence of adverse events. Pooled hazard ratios were calculated using a random-effects model based on log hazard ratios and standard error values. **Results**: Seven studies comprising approximately 774 patients were included in this analysis. The pooled hazard ratio was 0.80 (95% confidence interval, 0.42–1.53) for recurrence-free survival and 0.76 (95% confidence interval, 0.46–1.26) for progression-free survival, indicating no significant difference between gemcitabine and Bacillus Calmette–Guérin. However, gemcitabine was associated with a significantly lower incidence of adverse events, with a pooled odds ratio of approximately 0.48 (95% confidence interval, 0.27–0.86). Risk-of-bias assessment revealed that most randomized trials had “some concerns” based on the Risk of Bias 2 tool, whereas non-randomized studies were rated as having “moderate to serious” risk of bias according to the Risk of Bias in Non-randomized Studies of Interventions tool. **Conclusions**: Gemcitabine demonstrated an oncologic efficacy comparable to that of Bacillus Calmette–Guérin in terms of recurrence and progression outcomes while showing a substantially lower incidence of treatment-related toxicities.

## Simple Summary

Bladder cancer that has not invaded the muscle layer is usually treated with surgery followed by medication placed directly into the bladder to reduce the risk of cancer returning. The most commonly used treatment is Bacillus Calmette–Guérin (BCG), but it can cause side effects and is sometimes difficult to obtain due to supply shortages. Gemcitabine has been proposed as an alternative treatment, but it has been unclear whether it works as well as BCG. In this study, we combined results from multiple clinical studies to compare these two treatments. We found that gemcitabine was as effective as BCG in preventing cancer recurrence and progression, while causing fewer side effects. These findings may help doctors choose safer treatment options, especially for patients who cannot tolerate BCG or when BCG is unavailable.

## 1. Introduction

Intravesical instillation of Bacillus Calmette–Guérin (BCG) following transurethral resection of bladder tumor (TURBT) has long been recognized as the standard adjuvant therapy for patients with intermediate- and high-risk non-muscle-invasive bladder cancer (NMIBC) [1]. Both intravesical chemotherapy and BCG immunotherapy have demonstrated effectiveness in reducing tumor recurrence, with BCG additionally proven to lower the risk of disease progression [2,3]. Nevertheless, despite its established efficacy, BCG treatment is frequently constrained by significant local and systemic adverse effects, as well as periodic worldwide shortages of the vaccine supply [4,5]. These limitations have encouraged the exploration of alternative intravesical agents that could offer comparable oncologic outcomes while improving tolerability and treatment accessibility.

Many other chemotherapeutic agents, such as mitomycin C (MMC), gemcitabine, and epirubicin, have been used as intravesical adjuvant therapy post-TURBT as alternatives to BCG or as second-line therapy [6].

Many other chemotherapeutic agents, such as mitomycin C (MMC), gemcitabine, and epirubicin, have been used as intravesical adjuvant therapy following TURBT as alternatives to BCG or as second-line treatment options [6]. These intravesical chemotherapeutic agents aim to reduce the risk of tumor recurrence and progression by delivering cytotoxic drugs directly to the bladder mucosa. Compared with systemic chemotherapy, intravesical administration allows for high local drug concentrations while minimizing systemic toxicity. Among these agents, MMC and epirubicin have been widely used in clinical practice, while gemcitabine has gained increasing attention because of its favorable safety profile and promising oncologic outcomes in patients with NMIBC [7,8].

Intravesical gemcitabine was initially introduced as a novel therapeutic option for patients with BCG-refractory NMIBC by Dalbagni et al. Gemcitabine, a pyrimidine antimetabolite with both cytotoxic and immunomodulatory properties [9], has demonstrated promising results as an intravesical agent in both BCG-refractory and BCG-naïve NMIBC settings [10]. Several randomized controlled trials (RCTs) and cohort studies have compared gemcitabine with BCG following TURBT; however, their results remain inconsistent, with some studies reporting similar efficacy and others suggesting inferior oncologic outcomes but better tolerability with gemcitabine [10,11]. A previous meta-analysis, which included four RCTs and one case–control study [11], reported comparable efficacy between gemcitabine and BCG, whereas another meta-analysis incorporating four RCTs, one prospective study, and one case–control study also supported these findings [10].

Given the limitations of the existing evidence and the variability in study designs, patient populations, and treatment protocols among previous reports, the true comparative efficacy and safety of intravesical gemcitabine versus BCG remain uncertain. Furthermore, recent clinical studies, including RCTs and large-scale retrospective cohorts, have provided new data that were not incorporated in prior meta-analyses [12,13,14]. Therefore, we conducted an updated systematic review and meta-analysis to comprehensively evaluate and synthesize the current evidence comparing intravesical gemcitabine and BCG following TURBT in patients with NMIBC, focusing on key oncologic outcomes such as recurrence-free survival (RFS), progression-free survival (PFS), and the incidence of treatment-related adverse events (AEs).

## 2. Materials and Methods

This systematic review was registered in PROSPERO (CRD42023475252).

### 2.1. Literature Search

We conducted a systematic search of the PubMed, Embase, and Cochrane Library databases to identify relevant studies published up to September 2025. These databases were selected because they provide broad coverage of biomedical literature related to urologic oncology and intravesical therapy. The search strategy aimed to identify studies evaluating the efficacy and safety of intravesical gemcitabine compared with BCG after TURBT in patients with NMIBC.

This review included English-language comparative studies that evaluated intravesical gemcitabine and BCG administered after TURBT. Eligible studies were required to report at least one outcome of interest, including RFS, PFS, or treatment-related AEs. Both RCTs and observational comparative studies were considered eligible. Studies were excluded if they were conference abstracts, review articles, editorials, case reports, or single-arm studies without a comparator group. Studies evaluating combination regimens or treatment strategies unrelated to intravesical gemcitabine or BCG were also excluded.

The search strategy incorporated controlled vocabulary terms and free-text keywords to maximize sensitivity. Specifically, MeSH terms in PubMed and Emtree terms in Embase were combined with keywords related to bladder cancer, BCG, and gemcitabine using Boolean operators such as “AND” and “OR”. The detailed search strategies for each database are provided in the Supplementary Information.

Two reviewers independently screened titles and abstracts according to predefined inclusion and exclusion criteria. Studies considered potentially eligible underwent full-text review to determine final inclusion. Any disagreements between reviewers were resolved through discussion and consensus, with consultation from a third reviewer when necessary.

### 2.2. Trial Inclusion and Exclusion Criteria

Following the PRISMA guidelines and applying the PICOS framework [15], we conducted a systematic review and meta-analysis to evaluate the comparative efficacy and safety of intravesical gemcitabine and BCG after TURBT in patients with NMIBC. The population consisted of patients diagnosed with NMIBC who underwent TURBT as the initial treatment. The intervention of interest was intravesical gemcitabine instillation, whereas the comparator was intravesical BCG therapy administered according to the protocols used in the included studies.

The outcomes of interest included RFS, PFS, and the incidence of treatment-related AEs. RFS and PFS were selected as primary oncologic outcomes because they reflect tumor recurrence and disease progression following intravesical therapy. The incidence of AEs was evaluated to assess the safety and tolerability of the two treatment strategies.

Studies were considered eligible if they directly compared intravesical gemcitabine and BCG following TURBT and reported extractable outcome data for at least one of the predefined endpoints. Both RCTs and observational comparative studies were included to capture the available clinical evidence. Reviews, commentaries, editorials, conference abstracts, case reports, and single-arm studies without a comparator group were excluded. Studies lacking extractable outcome data or evaluating treatment regimens unrelated to intravesical gemcitabine or BCG were also excluded.

The primary endpoints of this meta-analysis were RFS and PFS, while the secondary endpoint was the incidence of treatment-related AEs, defined as the overall incidence of AEs reported in the included studies.

### 2.3. Data Extraction

Two authors independently extracted data from all eligible studies using a predefined data collection template developed for this systematic review. Extracted information included general study characteristics such as the first author, publication year, country of origin, study design, and total sample size. The number of participants allocated to each treatment group (gemcitabine vs. BCG) was also recorded.

Baseline patient characteristics were collected when available, including patient age and tumor risk classification. These variables were recorded to evaluate potential differences in baseline clinical characteristics among the included studies. Treatment-related variables were also extracted to allow comparison of treatment protocols across studies. These variables included the dosage of intravesical gemcitabine or BCG, the number of intravesical instillations administered during treatment, the treatment schedule when reported, and the duration of follow-up.

Clinical outcome data were extracted for all predefined endpoints of interest. These included RFS, PFS, and the incidence of treatment-related AEs. When available, effect estimates such as HRs or the number of events in each treatment group were collected to enable quantitative synthesis in the meta-analysis.

In addition, information regarding funding sources and potential conflicts of interest was recorded for each study to provide transparency regarding potential sources of bias. Any discrepancies between the two reviewers during the data extraction process were resolved through discussion and consensus. When necessary, the original articles were re-examined to verify the extracted information and ensure the accuracy and reliability of the collected data.

### 2.4. Study Quality Assessments and Quality of Evidence

To assess the methodological quality of the included studies, we used different risk-of-bias assessment tools according to study design. For RCTs, we applied the RoB 2 tool, whereas for non-randomized studies, we used the ROBINS-I tool [16,17]. These design-specific tools were used because potential sources of bias differ between randomized and observational studies. Two reviewers independently performed the quality assessment, and any disagreements were resolved through discussion and consensus.

For randomized trials, the RoB 2 tool evaluated five domains: the randomization process, deviations from intended interventions, missing outcome data, measurement of outcomes, and selection of reported results [17]. Each domain was assessed using the signaling questions provided in the RoB 2 framework, and an overall judgment was assigned for each study. The final judgments were categorized as low risk of bias, some concerns, or high risk of bias.

For non-randomized studies, the ROBINS-I tool assessed bias across three phases: pre-intervention, at-intervention, and post-intervention [16]. The pre-intervention phase addressed confounding and participant selection. The at-intervention phase evaluated the classification of interventions. The post-intervention phase assessed deviations from intended interventions, missing data, outcome measurement, and selective reporting. Each study was categorized as having low, moderate, serious, or critical risk of bias.

We also evaluated the overall certainty of evidence for each pooled outcome using the GRADE approach [18]. This framework considers study design, risk of bias, precision, inconsistency, indirectness, and publication bias. Based on these domains, the level of evidence was categorized as high, moderate, low, or very low. GRADEpro software (https://gradepro.org, McMaster University, Hamilton, ON, Canada) was used to generate the summary of findings and present the evidence profiles for each outcome.

### 2.5. Statistical Analyses

The authors extracted data on RFS, PFS, and the number of AEs from each included study to evaluate treatment outcomes between intravesical gemcitabine and BCG. These outcomes were selected because they represent clinically relevant endpoints for assessing both oncologic efficacy and treatment safety in NMIBC.

For time-to-event outcomes such as RFS and PFS, HRs with corresponding 95% CIs were extracted when reported in the original studies. If HRs were not directly provided, they were estimated from available survival data, including Kaplan–Meier curves, using established statistical methods [19]. In such cases, survival probabilities and event numbers were approximated from the published curves to reconstruct HR estimates, allowing inclusion of studies that reported survival outcomes graphically. For dichotomous outcomes such as treatment-related AEs, ORs and 95% CIs were calculated using stratified 2 × 2 contingency tables. Event data were extracted for both treatment groups and pooled to compare the incidence of AEs between the gemcitabine and BCG groups.

Statistical heterogeneity was assessed using the Cochrane Q test and the I^2^ statistic. Significant heterogeneity was defined as *p* < 0.05 for the Q test or I^2^ > 50% [20]. A random-effects model based on the DerSimonian–Laird method was applied to pool effect estimates, accounting for both within-study and between-study variability [21]. Sensitivity analyses were performed using a leave-one-out approach to assess the robustness of pooled estimates. Publication bias was evaluated using funnel plots, where visual symmetry suggested the absence of small-study effects.

All statistical analyses were performed using Review Manager (RevMan) v5.4 (the Cochrane Collaboration, Copenhagen, Denmark), and two-tailed *p*-values < 0.05 were considered statistically significant.

## 3. Results

### 3.1. Systematic Review Process

**Figure 1:**
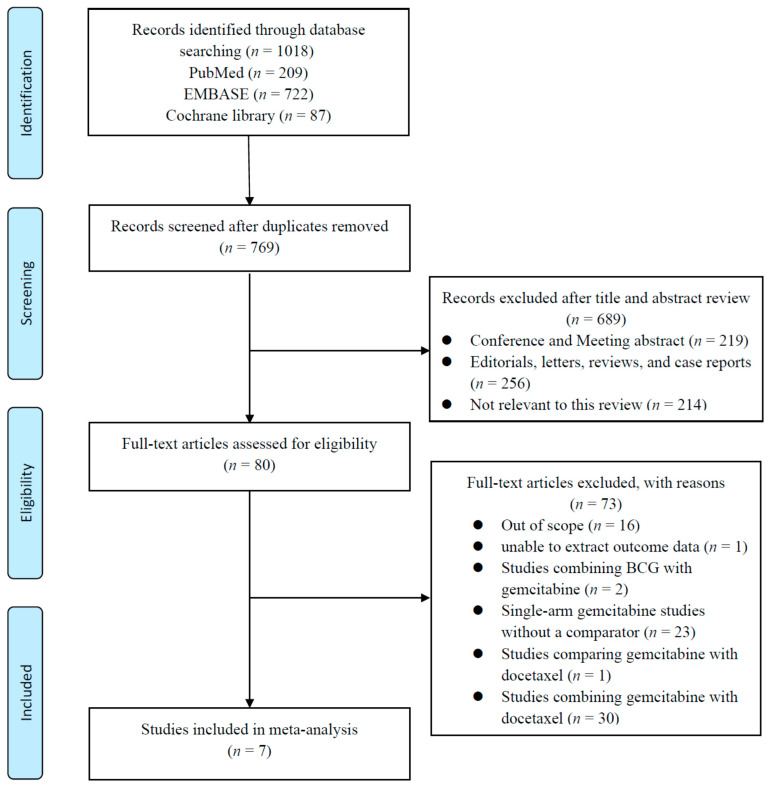
Flowchart of preferred reporting items for systematic reviews and meta-analysis (PRISMA). BCG, Bacille Calmette–Guérin.

The study selection process followed the predefined systematic review protocol described in Section 2. After applying the eligibility criteria to the records identified through database searches, studies were screened based on titles and abstracts, followed by full-text assessment to determine final eligibility. The detailed selection process and reasons for exclusion are summarized in the PRISMA flow diagram (Figure 1), which illustrates each stage of the screening and study selection process. The PRISMA 2020 checklist is provided in Supplementary Table S2.

The initial database search identified 1018 records, including 209 from PubMed, 722 from Embase, and 87 from the Cochrane Library. After removing duplicate entries, 769 studies remained for title and abstract screening. After the initial screening, 689 studies were excluded because they were irrelevant to the study topic or did not meet the predefined inclusion criteria. The full texts of 80 articles were subsequently reviewed in detail to determine eligibility based on the predefined selection criteria. Finally, seven studies met all eligibility criteria and were included in the final meta-analysis [6,10,11,12,22,23,24].

A detailed summary of the included studies is presented in Table 1. Among these studies, four were RCTs, whereas the remaining studies were retrospective observational studies. All included studies compared intravesical gemcitabine and BCG following TURBT and evaluated their effects on oncologic outcomes and treatment-related adverse events in patients with NMIBC. These studies provided the data used for the quantitative synthesis performed in this meta-analysis and served as the basis for the pooled analyses presented in Section 3. Overall, the included studies provided a comparative evaluation of the efficacy and safety of intravesical gemcitabine and BCG in the adjuvant treatment of NMIBC after TURBT.

### 3.2. Comparison of Recurrence-Free Survival Between the Gemcitabine and BCG Groups

**Figure 2:**
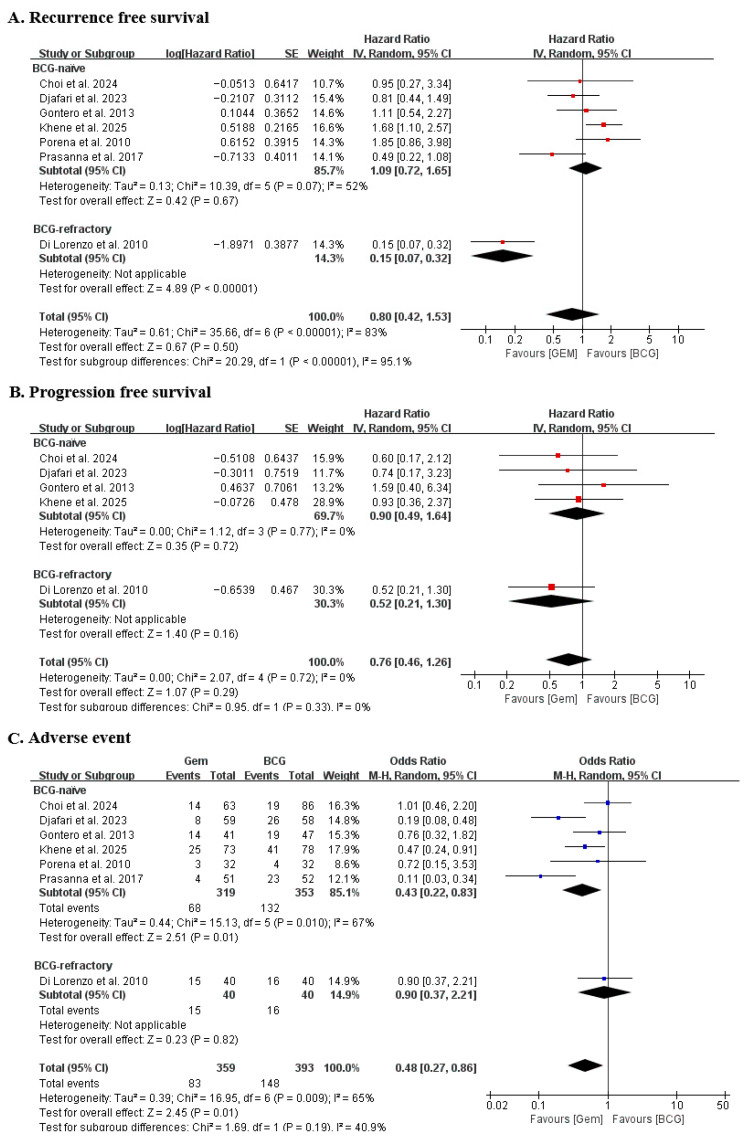
Forest plots of outcomes between gemcitabine and Bacillus Calmette-Guérin groups (**A**) recurrence-free survival, (**B**) progression-free survival, and (**C**) adverse event. SE, standard error; CI: confidence interval; GEM, gemcitabine; BCG, Bacillus Calmette–Guérin; df, degrees of freedom [6,10,11,12,22,23,24].

Seven studies were included in the pooled RFS analyses. In the overall analysis, the combined HR was 0.80 (95% CI: 0.42–1.53; *p* = 0.51), indicating no significant difference in recurrence risk between the gemcitabine and BCG groups. However, substantial heterogeneity was observed (I^2^ = 83%, *p* < 0.00001) (Figure 2).

To explore potential sources of heterogeneity, subgroup analyses were performed according to prior BCG exposure. In the BCG-naïve subgroup, no significant difference in recurrence-free survival was observed between treatments (HR 1.09, 95% CI: 0.72–1.65; I^2^ = 52%). In contrast, in the BCG-refractory subgroup, gemcitabine demonstrated a significant reduction in recurrence risk (HR 0.15, 95% CI: 0.07–0.32). The test for subgroup differences was statistically significant (*p* < 0.00001), indicating that prior BCG exposure significantly modified the treatment effect. These findings suggest that pooling naïve and refractory populations may obscure clinically meaningful differences.

### 3.3. Comparison of Progression-Free Survival Between the Gemcitabine and BCG Groups

Five studies were included in the pooled PFS analyses. The overall pooled HR was 0.76 (95% CI: 0.46–1.26; *p* = 0.29), indicating that there was no statistically significant difference in progression risk between the gemcitabine and BCG treatment groups. Assessment of heterogeneity showed no significant variability among the included studies (I^2^ = 0%), suggesting a high level of consistency in the reported progression outcomes across studies (Figure 2).

Subgroup analysis according to prior BCG exposure was performed to explore potential differences in treatment effects among different patient populations. The analysis showed no statistically significant interaction between subgroups (*p* = 0.33), indicating that the treatment effect was generally consistent regardless of prior BCG exposure. In the BCG-naïve subgroup, the pooled HR was 0.90 (95% CI: 0.49–1.64), whereas in the BCG-refractory subgroup the HR was 0.52 (95% CI: 0.21–1.30). Although the point estimate in the BCG-refractory subgroup suggested a trend toward a lower risk of progression with gemcitabine, the confidence interval crossed unity and did not reach statistical significance. Overall, these results suggest that progression outcomes were broadly comparable between the two treatments across different patient populations.

### 3.4. Comparison of Adverse Events Between the Gemcitabine and BCG Groups

Seven studies were included in the pooled analysis of treatment-related adverse events. Overall, gemcitabine was associated with a significantly lower incidence of AEs compared with BCG (OR 0.48, 95% CI: 0.27–0.86; *p* = 0.01) (Figure 2). These results indicate that patients receiving intravesical gemcitabine experienced fewer treatment-related adverse events than those treated with BCG. However, moderate heterogeneity was observed among the included studies (I^2^ = 65%), suggesting some variability in the reported incidence of adverse events across studies.

Subgroup analyses were performed according to prior BCG exposure to explore potential differences in safety outcomes between patient populations. In the BCG-naïve population, gemcitabine demonstrated a significantly lower incidence of AEs compared with BCG (OR 0.43, 95% CI: 0.22–0.83). In contrast, no statistically significant difference in AE incidence was observed in the BCG-refractory subgroup (OR 0.90, 95% CI: 0.37–2.21). The test for subgroup differences was not statistically significant (*p* = 0.19), indicating that the overall reduction in adverse events associated with gemcitabine was generally consistent across subgroups, although the magnitude of the effect varied between populations.

### 3.5. Quality Assessment and Qualitative Risk of Bias

The quality assessment of the included studies, conducted using the ROBINS-I (v2016) and RoB 2 tools (v2019), is summarized in Supplementary Figures S1 and S2, respectively. All non-randomized studies were judged to have an overall moderate-to-serious risk of bias, primarily due to potential confounding factors and variations in treatment selection across study populations. In retrospective studies, differences in patient characteristics and treatment indications may influence treatment allocation and outcome assessment, which could contribute to residual confounding.

Among the four RCTs, three were rated as having “some concerns,” whereas one was assessed as having a high risk of bias. The higher risk of bias was mainly related to the absence of blinding and incomplete follow-up data reported in the study. These methodological limitations were considered when interpreting the overall findings of the meta-analysis.

A summary of findings table was developed using the GRADE framework to assess the certainty of evidence for each outcome (Table 2). For RFS, the certainty of evidence was rated as very low owing to potential confounding factors, imbalance in follow-up duration, and unblinded study designs, together with considerable heterogeneity among the included studies. Similarly, the certainty of evidence for PFS was also judged to be very low, mainly because of methodological limitations and imprecision of the effect estimates. In contrast, the certainty of evidence for AEs was considered high, as the results were relatively consistent across studies and were based on clearly defined and objectively measured outcomes, demonstrating a strong association favoring gemcitabine over BCG.

Funnel plots were constructed to evaluate potential publication bias for the analyzed outcomes. Visual inspection of the plots demonstrated a generally symmetrical distribution of the included studies, suggesting no significant evidence of publication bias across the analyzed outcomes (Supplementary Figure S2).

Leave-one-out sensitivity analyses were performed using a DerSimonian–Laird random-effects model to examine the robustness of the pooled estimates. Sequential omission of individual studies did not materially alter the overall conclusions for RFS, PFS, or AEs, indicating that the results of the meta-analysis were stable and not disproportionately influenced by any single study (Supplementary Table S1). 

## 4. Discussion

The present meta-analysis demonstrated that intravesical gemcitabine provided oncologic outcomes comparable to those of BCG after TURBT in patients with NMIBC. Pooled analyses showed no significant difference in RFS (HR = 0.80; 95% CI: 0.42–1.53) and PFS (HR = 0.76; 95% CI: 0.46–1.26) between the two treatment groups. However, gemcitabine was associated with a significantly lower incidence of treatment-related AEs than BCG (OR, 0.48; 95% CI: 0.27–0.86). These findings suggest that while both intravesical agents demonstrate similar efficacy in preventing recurrence and progression, gemcitabine offers a more favorable safety profile.

The comparable oncologic outcomes observed in this analysis indicate that gemcitabine may provide adequate tumor control in patients with NMIBC following TURBT. At the same time, the lower incidence of treatment-related AEs suggests that gemcitabine may be better tolerated than BCG in clinical practice. Taken together, these findings support the potential role of intravesical gemcitabine as a viable therapeutic alternative, particularly for patients who are unable to tolerate BCG or in situations where BCG availability is limited. In addition, the improved safety profile may contribute to better treatment adherence and overall patient tolerability during intravesical therapy.

Gemcitabine is a pyrimidine antimetabolite that inhibits DNA synthesis by blocking the conversion of deoxycytidine triphosphate, thereby preventing tumor cell proliferation [25]. Beyond its cytotoxic effect, gemcitabine also exhibits immunomodulatory activity by enhancing antigen presentation and inducing tumor-specific immune responses within the bladder microenvironment [26]. This dual mechanism, involving both direct antitumor activity and modulation of the immune response, may contribute to its therapeutic effectiveness in the management of NMIBC. When administered intravesically, gemcitabine demonstrates pharmacokinetic properties that are favorable for local treatment. Its relatively high molecular weight and polarity limit systemic absorption, allowing for sustained exposure within the bladder while minimizing systemic toxicity [27]. This characteristic is particularly important in intravesical therapy, where maintaining high local drug concentrations with minimal systemic exposure is essential for achieving effective tumor control while preserving patient safety. Several preclinical and clinical studies have shown that gemcitabine penetrates the urothelial layer effectively, achieving high local concentrations that are cytotoxic to residual tumor cells following TURBT [27,28]. These pharmacologic characteristics make gemcitabine a promising intravesical agent capable of providing durable oncologic control with a relatively favorable toxicity profile compared with BCG or MMC [10,11,29]. In addition to clinical and pharmacologic studies, mathematical modeling approaches have also been used to better understand the dynamics of bladder cancer treatment and immune–tumor interactions during intravesical therapy. Previous studies have explored treatment dynamics of intravesical therapies such as BCG and chemotherapy using multiscale mathematical models, providing theoretical insights into tumor–immune interactions and treatment response patterns during intravesical therapy [22,23].

The comparable efficacy observed between gemcitabine and BCG suggests that gemcitabine possesses sufficient antitumor activity to prevent the recurrence and progression of NMIBC, despite its different pharmacologic mechanisms. These findings are consistent with previous RCTs and meta-analyses that reported oncologic outcomes for gemcitabine comparable to BCG, along with a more favorable safety profile [10,11,13,30]. The comparable oncologic outcomes observed in this analysis indicate that gemcitabine may provide adequate disease control in patients with NMIBC following TURBT. Considering the significantly lower incidence of adverse events, intravesical gemcitabine may be particularly beneficial for older patients, those with multiple comorbidities, or individuals who are unable to tolerate BCG therapy [10,11]. In clinical practice, treatment-related toxicity often influences treatment adherence and continuation of intravesical therapy; therefore, a treatment option with improved tolerability may contribute to better patient compliance and overall treatment outcomes. Furthermore, gemcitabine represents a practical and accessible alternative during ongoing global BCG shortages. In situations where BCG availability is limited, the use of intravesical gemcitabine may provide an effective therapeutic option while maintaining oncologic efficacy and minimizing treatment-related toxicity [5]. These considerations highlight the potential role of gemcitabine as an alternative intravesical therapy in selected patient populations with NMIBC.

This meta-analysis has several strengths that contribute to the validity and relevance of the findings. First, the inclusion of recent studies ensured that the analysis reflected updated clinical evidence and provided a current perspective on the comparative efficacy and safety of intravesical gemcitabine and BCG. By incorporating recently published data, this study offers a more comprehensive evaluation of the available literature and reflects current treatment practices for NMIBC. Unlike some previous meta-analyses, the present study demonstrated that intravesical gemcitabine was significantly safer than BCG, showing a markedly lower incidence of treatment-related AEs while maintaining comparable oncologic efficacy [10,11]. This finding is particularly important because treatment-related toxicity can significantly affect patient adherence and overall treatment tolerability in clinical practice. In addition, the use of clearly defined oncologic outcomes, including RFS and PFS, allowed for a consistent evaluation of treatment efficacy across the included studies. The inclusion of adverse event data further enabled a balanced assessment of both oncologic outcomes and treatment safety. Taken together, these methodological strengths enhance the reliability of the findings and highlight intravesical gemcitabine as a promising alternative to BCG for the management of NMIBC.

However, this study has several limitations. Most studies included in this meta-analysis were RCTs (*n* = 4), and the remaining studies were non-randomized (*n* = 3). Although several RCTs were included, their methodological quality was restricted by small sample sizes, lack of blinding, and unclear allocation, and together with the inherent bias of non-randomized studies, the overall evidence remains low, which limits the generalizability of the results. The significant heterogeneity observed among the studies indicates variability in treatment protocols, follow-up durations, and patient populations, which may have affected the pooled estimates. BCG regimens varied across the included studies in terms of strain, dosage, and instillation schedules, with reported doses ranging from 12.5 mg to 27 mg and up to 5 × 10^8^ CFU. Maintenance protocols were also inconsistently applied. Such differences may directly influence toxicity profiles and could have contributed to the heterogeneity observed in the adverse event analysis (I^2^ = 65%). Given the limited number of studies and variability in reporting, stratified analyses by BCG dose or strain were not feasible, and this should be considered when interpreting the safety outcomes. Furthermore, differences in maintenance therapy adherence between the treatment groups could have influenced the outcomes. The number of included studies was fewer than 10 for all outcomes, limiting the reliability of funnel plot–based assessments of publication bias. Formal evaluation of small-study effects is generally underpowered with such a limited number of studies. Given the relatively small number of included studies, the DerSimonian–Laird estimator may underestimate between-study variance, and therefore the results should be interpreted cautiously. The interpretation of adverse event outcomes should be made cautiously because definitions and reporting of AEs varied across the included studies. In particular, the severity grading of adverse events (e.g., grade 1–2 vs. grade ≥3) and differentiation between local and systemic toxicities were not consistently reported, which precluded stratified quantitative analyses. To address these limitations, future research should focus on large-scale, multicenter, RCTs with longer follow-up periods to verify these findings and assess long-term outcomes.

## 5. Conclusions

The results of the present study suggest that intravesical gemcitabine provides oncologic outcomes similar to those of BCG after TURBT in patients with NMIBC. The oncologic outcomes (RFS and PFS) were comparable between the two treatments; however, gemcitabine was associated with a significantly lower incidence of AEs. These findings highlight the potential of gemcitabine as an alternative to BCG, particularly in patients who cannot tolerate BCG or in settings where the supply of BCG is limited. However, the certainty of evidence for oncologic outcomes remains very low, and substantial clinical and statistical heterogeneity was observed across studies. These findings should, therefore, be interpreted with caution and considered hypothesis-generating. Further well-designed, adequately powered randomized controlled trials are needed to clarify the comparative effectiveness of intravesical gemcitabine and BCG.

## Figures and Tables

**Table 1:** Characteristics of studies included in this meta-analysis.

Study	Country	Design of Study	Tumor Characteristics	No. GEM	No. BCG	Age (Years)		GEM		BCG		Conflict of Interest
						Gem	BCG	Dosag	No. of Instillations	Dosage	No. of Instillations	
Choi et al. (2024) [10]	South Korea	Retrospective multicenter cohort	Intermediate- and high-risk NMIBC, BCG-naïve	63	86	72.8 ± 9.8	70.6 ± 9.2	2000 mg in 50 mL saline	10	12.5 mg in 100 mL saline	6.4	None
Di Lorenzo et al. (2010) [22]	Italy	RCT	High-risk NMIBC, all with BCG failure after 1 course	40	40	69.3 ± 8.4	71.4 ± 7.9	2000 mg in 50 mL saline	15	12.5 mg in 100 mL saline	15	None
Djafari et al. (2023) [11]	Iran	RCT	Intermediate-risk NMIBC, BCG-naïve	59	58	63.95 ± 10.5	62.36 ± 10.9	1000 mg in 50 mL saline	6	12.5 mg in 100 mL saline	6	None
Gontero et al. (2013) [23]	Italy and Germany	RCT	Intermediate-risk NMIBC, BCG-naïve	61	59	67.4 ± 9.4	67.5 ± 9.8	2000 mg in 50 mL saline	15	27 mg in 50 mL saline	15	None
Khene et al. (2025) [12]	USA	Retrospective cohort	Low-grade intermediate-risk NMIBC, BCG-naïve	73	68	72 (IQR, 62–78)	68 (IQR, 62–75)	2000 mg in 50 mL saline	14	12.5 mg in 100 mL saline	12	None
Porena et al. (2010) [24]	Italy	RCT	High-risk superficial bladder cancer, BCG-naïve	32	32	70.2 ± 5.5	68.7 ± 10.2	2000 mg in 50 mL saline	27	5 × 10^8^ CFU in 50 mL saline	27	None
Prasanna et al. (2017) [6]	Australia	Retrospective cohort	NMIBC (CIS, pTa, pT1)	51	52	78	77	2000 mg in 50 mL saline	10.5–12	5 × 10^8^ CFU in 50 mL saline	10.5–12	None

GEM, gemcitabine; BCG, Bacillus Calmette–Guérin; NMIBC, non-muscle-invasive bladder cancer; RCT, randomized controlled trial; IQR, interquartile range; CFU, colony-forming unit.

**Table 2:** Grading of recommendations, assessments, developments, and evaluation quality assessment of evidence of each comparison.

Certainty Assessment							Number of Patients		Effect		Certainty	Importance
Number of Studies	Study Design	Risk of Bias	Inconsistency	Indirectness	Imprecision	Other Considerations	BCG	GEM	Relative (95% CI)	Absolute (95% CI)		
Recurrence-free survival												
7	RCTs and cohort studies	Serious ^a^	Serious ^b^	Not serious	Serious ^c^	None	−/0	−/0	HR, 0.80 (0.42 to 1.53)	1 fewer per 1000 (from 2 fewer to 0 fewer)	Very low ^a,b,c^	Critical
Progression-free survival												
5	RCTs and cohort studies	Serious ^a^	Not serious	Not serious	Serious ^c^	None	−/0	−/0	HR, 0.76 (0.46 to 1.26)	1 fewer per 1000 (from 1 fewer to 0 fewer)	Very low ^a,c^	Critical
Adverse events												
7	RCTs and cohort studies	Not serious	Not serious	Not serious	Serious ^c^	Strong association	83/359 (23.1%)	148/393 (37.7%)	OR, 0.48 (0.27 to 0.86)	152 fewer per 1000 (from 236 fewer to 35 fewer)	High ^c^	Critical

^a^ Confounding, follow-up imbalance, unblinded design. ^b^ *p* < 0.05 and I^2^ = 83%. ^c^ The result includes 0. BCG, Bacillus Calmette–Guérin; GEM, gemcitabine; CI: confidence interval; RCT, randomized controlled trial; HR, hazard ratio; OR, odds ratio.

## Data Availability

Data sharing is not applicable to this article as no new data were created or analyzed beyond those available in published studies.

## Supplementary Materials

The following supporting information can be downloaded at: https://www.mdpi.com/article/10.3390/cancers18060990/s1, Table S1: Leave-One-Out Sensitivity Analyses Using a Random-Effects Model; Table S2: PRISMA 2020 Checklist; Figure S1: Plot of risk of bias; Figure S2: Summary of risk of bias.

## Abbreviations

The following abbreviations are used in this manuscript:

AE	Adverse Event
BCG	Bacillus Calmette–Guérin
CI	Confidence Interval
GRADE	Grading of Recommendations, Assessment, Development, and Evaluation
HR	Hazard Ratio
IQR	Interquartile Range
MMC	Mitomycin C
NMIBC	Non-Muscle-Invasive Bladder Cancer
OR	Odds Ratio
PFS	Progression-Free Survival
PICOS	Population, Intervention, Comparator, Outcome, Study Design
PRISMA	Preferred Reporting Items for Systematic Reviews and Meta-Analyses
PROSPERO	International Prospective Register of Systematic Reviews
RCT	Randomized Controlled Trial
ROBINS-I	Risk Of Bias In Non-randomized Studies of Interventions
RoB 2	Risk of Bias 2 Tool
RFS	Recurrence-Free Survival
TURBT	Transurethral Resection of Bladder Tumor
