# Supplementary material for: Efficacy and Toxicity in Scheduled Intravesical Gemcitabine Versus Bacillus Calmette–Guérin for Bladder Cancer: A Systematic Review and Meta-Analysis
Source: Cancers (Basel). 2026 Mar 18;18(6):990. doi: 10.3390/cancers18060990 (PMC13024882; doi:10.3390/cancers18060990)
Supplement: Supplementary file 1 [file cancers-18-00990-s001.zip › cancers-4180409-supplementary.pdf]

Search terms

1. Pubmed

((("bladder malignanc*"[Title/Abstract] OR ("urinary bladder neoplasms"[MeSH Terms] OR ("bladder cancer*"[Title/Abstract] OR "bladder neoplasm*"[Title/Abstract] OR "bladder tumo*"[Title/Abstract] OR "urinary bladder neoplasm*"[Title/Abstract])) OR ("carcinoma, transitional cell"[MeSH Terms] OR "transitional cell carcinoma*"[Title/Abstract]) OR "transitional cell cancer*"[Title/Abstract] OR "urothelial cancer*"[Title/Abstract] OR "urothelial carcinoma*"[Title/Abstract] OR "urothelial malignanc*"[Title/Abstract] OR "bladder carcinoma*"[Title/Abstract] OR "bladder papillom*"[Title/Abstract] OR "urothelial papillom*"[Title/Abstract] OR "transitional cell neoplasm*"[Title/Abstract] OR "transitional cell tumo*"[Title/Abstract] OR "urothelial tumo*"[Title/Abstract] OR "transitional cell malignanc*"[Title/Abstract] OR "transitional cell papillom*"[Title/Abstract]) AND ("mycobacterium bovis"[MeSH Terms] OR ("mycobacterium bovis"[Title/Abstract] OR "calmette guerin bacillus"[Title/Abstract] OR "mycobacterium bovis BCG"[Title/Abstract] OR "BCG"[Title/Abstract]) OR "bacille calmette guerin"[Title/Abstract] OR "bacillus calmette guerin"[Title/Abstract]) AND ("gemcitabine"[MeSH Terms] OR "gemcitabine"[Title/Abstract] OR "GEM"[Title/Abstract])) NOT ("animals"[MeSH Terms] NOT "humans"[MeSH Terms])) AND ((english[Filter]) AND (alladult[Filter]))

2. EMBASE

((('bladder cancer'/exp OR ('bladder malignanc*':ab,ti OR 'bladder cancer*':ab,ti)) OR ('bladder tumor'/exp OR ('bladder neoplasm*':ab,ti OR 'bladder tumo*':ab,ti OR 'urinary bladder neoplasm*':ab,ti)) OR ('transitional cell carcinoma'/exp OR ('transitional cell carcinoma*':ab,ti OR 'transitional cell cancer*':ab,ti OR 'urothelial cancer*':ab,ti OR 'urothelial carcinoma*':ab,ti OR 'urothelial malignanc*':ab,ti)) OR ('bladder carcinoma'/exp OR 'bladder carcinoma*':ab,ti) OR ('bladder papilloma'/exp OR 'bladder papillom*':ab,ti) OR ('urothelial papilloma'/exp OR 'urothelial papillom*':ab,ti) OR ('urothelial tumor'/exp OR ('transitional cell neoplasm*':ab,ti OR 'transitional cell tumo*':ab,ti OR 'urothelial tumo*':ab,ti)) OR 'transitional cell malignanc*':ab,ti OR 'transitional cell papillom*':ab,ti) AND (('mycobacterium bovis'/exp OR

'mycobacterium bovis':ab,ti) OR (('mycobacterium bovis'/exp OR 'mycobacterium bovis':ab,ti) OR ('calmette guerin bacillus':ab,ti OR 'mycobacterium bovis bcg':ab,ti OR bcg:ab,ti OR 'bacille calmette guerin':ab,ti OR 'bacillus calmette guerin':ab,ti))) AND (('gemcitabine'/exp OR gemcitabine:ab,ti) OR ('gem'/exp OR gem:ab,ti))) NOT ('animal'/exp NOT 'human'/exp) AND [english]/lim AND ([adult]/lim OR [young adult]/lim OR [middle aged]/lim OR [aged]/lim OR [very elderly]/lim)

3. Cochrane library

((((bladder next malignanc*):ab,ti OR ([mh "Urinary Bladder Neoplasms"] OR ((bladder next cancer*):ab,ti OR (bladder next neoplasm*):ab,ti OR (bladder next tumo*):ab,ti OR (urinary next bladder next neoplasm*):ab,ti)) OR ([mh "Carcinoma, Transitional Cell"] OR (transitional next cell next carcinoma*):ab,ti) OR (transitional next cell next cancer*):ab,ti OR (urothelial next cancer*):ab,ti OR (urothelial next carcinoma*):ab,ti OR (urothelial next malignanc*):ab,ti OR (bladder next carcinoma*):ab,ti OR (bladder next papillom*):ab,ti OR (urothelial next papillom*):ab,ti OR (transitional next cell next neoplasm*):ab,ti OR (transitional next cell next tumo*):ab,ti OR (urothelial next tumo*):ab,ti OR (transitional next cell next malignanc*):ab,ti OR (transitional next cell next papillom*):ab,ti) AND (([mh "Mycobacterium bovis"] OR ("mycobacterium bovis":ab,ti OR "calmette guérin bacillus":ab,ti OR "mycobacterium bovis BCG":ab,ti OR BCG:ab,ti)) OR "bacille calmette guérin":ab,ti OR "bacillus calmette guérin":ab,ti) AND (([mh Gemcitabine] OR gemcitabine:ab,ti) OR GEM:ab,ti)) NOT ([mh Animals] NOT [mh Humans])) AND [mh Adult]

A. Recurrence free survival

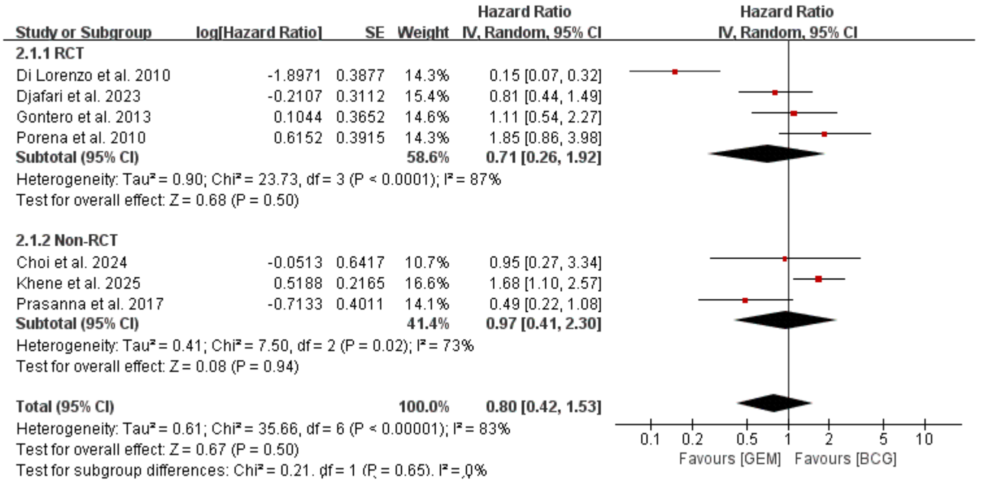

B. Progression free survival

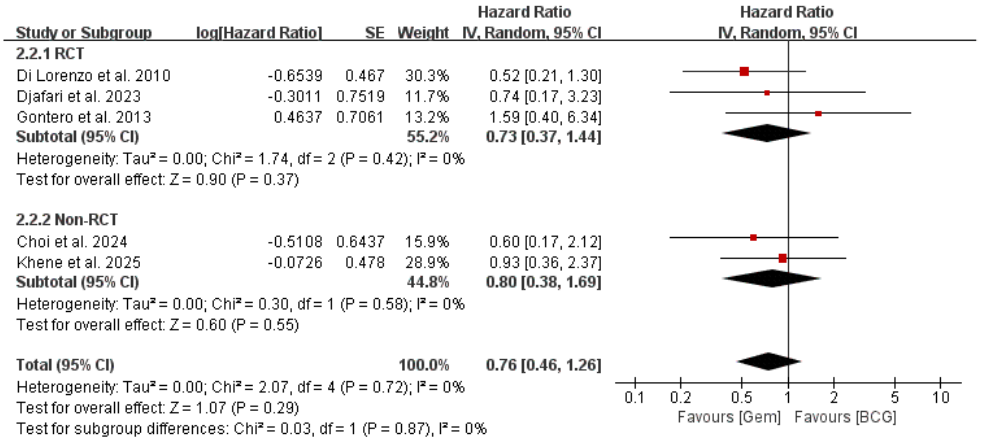

C. Adverse event

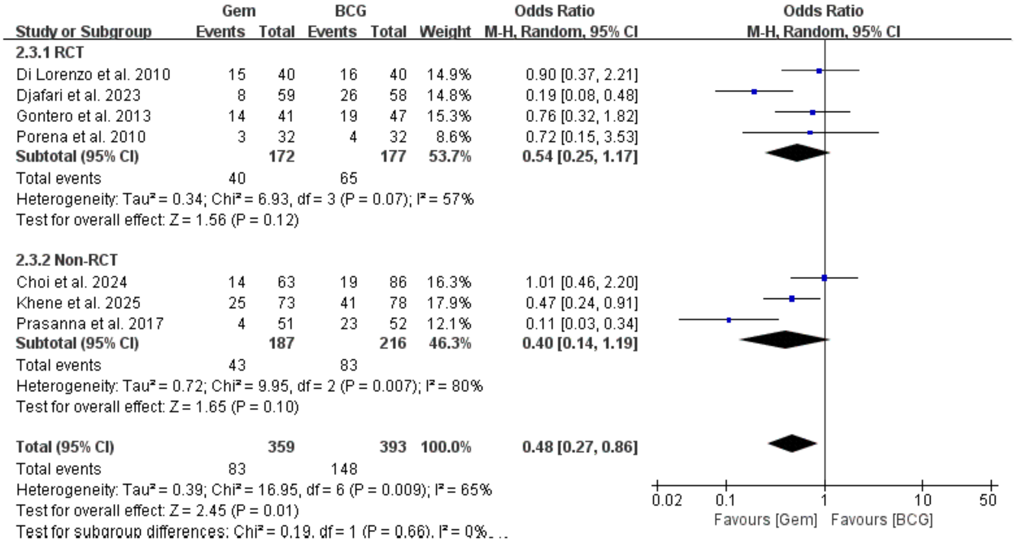

Supplementary Figure S1. Subgroup analysis of recurrence-free survival stratified by study design (randomized controlled trials vs. non-randomized studies).

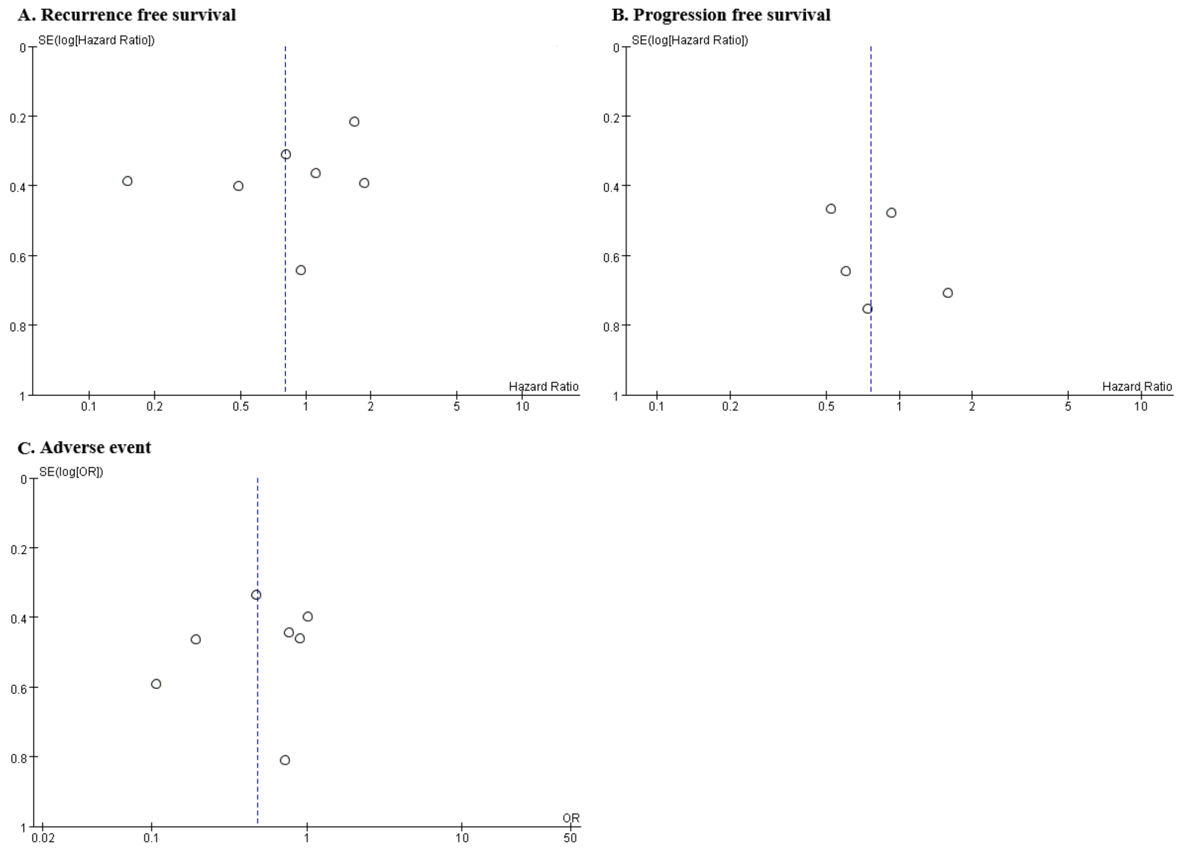

Supplementary Figure S2. Funnel plot of outcomes between the gemcitabine and Bacille Calmette–Guérin groups (**A**) recurrence-free survival**,** (**B**) progression-free survival, and (**C**) adverse event. SE, standard error; OR, odds ratio.

Supplementary Table S1. Leave-One-Out Sensitivity Analyses Using a Random-Effects Model.

A. Recurrence-Free Survival (RFS)

Analysis	Pooled HR	95% CI
Overall	0.80	0.42–1.53
Omit Di Lorenzo 2010	1.03	0.69–1.53
Omit Djafari 2023	0.81	0.38–1.72
Omit Gontero 2013	0.76	0.35–1.63
Omit Porena 2010	0.74	0.37–1.47
Omit Choi 2024	0.79	0.39–1.60
Omit Khene 2025	0.70	0.34–1.43
Omit Prasanna 2017	0.87	0.46–1.65

B. Progression-Free Survival (PFS)

Analysis	Pooled HR	95% CI
Overall	0.76	0.46–1.26
Omit Di Lorenzo 2010	0.83	0.50–1.37
Omit Djafari 2023	0.78	0.45–1.35
Omit Gontero 2013	0.71	0.42–1.22
Omit Choi 2024	0.79	0.47–1.33
Omit Khene 2025	0.81	0.48–1.36

C. Adverse Events (AEs)

Analysis	Pooled OR	95% CI
Overall	0.48	0.27–0.86
Omit Di Lorenzo 2010	0.44	0.25–0.79
Omit Djafari 2023	0.62	0.35–1.08
Omit Gontero 2013	0.53	0.29–0.95
Omit Porena 2010	0.46	0.26–0.82
Omit Choi 2024	0.47	0.26–0.84
Omit Khene 2025	0.55	0.31–0.96
Omit Prasanna 2017	0.59	0.33–1.03

Table S2. PRISMA 2020 Checklist.

Section and Topic	Item #	Checklist item	Location where item is reported
**TITLE**			
Title	1	Identify the report as a systematic review.	Title page
**ABSTRACT**			
Abstract	2	See the PRISMA 2020 for Abstracts checklist.	Abstract
**INTRODUCTION**			
Rationale	3	Describe the rationale for the review in the context of existing knowledge.	Introduction, paragraphs 1–3
Objectives	4	Provide an explicit statement of the objective(s) or question(s) the review addresses.	Introduction, final paragraph
**METHODS**			
Eligibility criteria	5	Specify the inclusion and exclusion criteria for the review and how studies were grouped for the syntheses.	Methods – Trial Inclusion and Exclusion Criteria
Information sources	6	Specify all databases, registers, websites, organisations, reference lists and other sources searched or consulted to identify studies. Specify the date when each source was last searched or consulted.	Methods – Literature Search
Search strategy	7	Present the full search strategies for all databases, registers and websites, including any filters and limits used.	Supplementary Information (Search terms)
Selection process	8	Specify the methods used to decide whether a study met the inclusion criteria of the review, including how many reviewers screened each record and each report retrieved, whether they worked independently, and if applicable, details of automation tools used in the process.	Methods – Literature Search
Data collection process	9	Specify the methods used to collect data from reports, including how many reviewers collected data from each report, whether they worked independently, any processes for obtaining or confirming data from study investigators, and if applicable, details of automation tools used in the process.	Methods – Data Extraction
Data items	10a	List and define all outcomes for which data were sought. Specify whether all results that were compatible with each outcome domain in each study were sought (e.g. for all measures, time points, analyses), and if not, the methods used to decide which results to collect.	Methods – Trial Inclusion and Exclusion Criteria / Data Extraction

Section and Topic	Item #	Checklist item	Location where item is reported
	10b	List and define all other variables for which data were sought (e.g. participant and intervention characteristics, funding sources). Describe any assumptions made about any missing or unclear information.	Methods – Data Extraction
Study risk of bias assessment	11	Specify the methods used to assess risk of bias in the included studies, including details of the tool(s) used, how many reviewers assessed each study and whether they worked independently, and if applicable, details of automation tools used in the process.	Methods – Study Quality Assessments and Quality of Evidence
Effect measures	12	Specify for each outcome the effect measure(s) (e.g. risk ratio, mean difference) used in the synthesis or presentation of results.	Methods – Statistical Analyses
Synthesis methods	13a	Describe the processes used to decide which studies were eligible for each synthesis (e.g. tabulating the study intervention characteristics and comparing against the planned groups for each synthesis (item #5)).	Methods – Trial Inclusion and Exclusion Criteria
	13b	Describe any methods required to prepare the data for presentation or synthesis, such as handling of missing summary statistics, or data conversions.	Methods – Statistical Analyses
	13c	Describe any methods used to tabulate or visually display results of individual studies and syntheses.	Methods – Statistical Analyses (forest plots, funnel plots)
	13d	Describe any methods used to synthesize results and provide a rationale for the choice(s). If meta-analysis was performed, describe the model(s), method(s) to identify the presence and extent of statistical heterogeneity, and software package(s) used.	Methods – Statistical Analyses
	13e	Describe any methods used to explore possible causes of heterogeneity among study results (e.g. subgroup analysis, meta-regression).	Methods – Statistical Analyses (heterogeneity, I^2)
	13f	Describe any sensitivity analyses conducted to assess robustness of the synthesized results.	Methods – Statistical Analyses
Reporting bias	14	Describe any methods used to assess risk of bias due to missing results in a synthesis (arising from reporting biases).	Methods –

Section and Topic	Item #	Checklist item	Location where item is reported
assessment			Statistical Analyses (funnel plots)
Certainty assessment	15	Describe any methods used to assess certainty (or confidence) in the body of evidence for an outcome.	Methods – Study Quality Assessments and Quality of Evidence (GRADE)
**RESULTS**			
Study selection	16a	Describe the results of the search and selection process, from the number of records identified in the search to the number of studies included in the review, ideally using a flow diagram.	Results – Systematic Review Process + Figure 1
	16b	Cite studies that might appear to meet the inclusion criteria, but which were excluded, and explain why they were excluded.	Results – Systematic Review Process
Study characteristics	17	Cite each included study and present its characteristics.	Results – Table 1
Risk of bias in studies	18	Present assessments of risk of bias for each included study.	Results – Quality Assessment and Qualitative Risk of Bias (Figures 4–5)
Results of individual studies	19	For all outcomes, present, for each study: (a) summary statistics for each group (where appropriate) and (b) an effect estimate and its precision (e.g. confidence/credible interval), ideally using structured tables or plots.	Results – Figures 2A–C
Results of syntheses	20a	For each synthesis, briefly summarise the characteristics and risk of bias among contributing studies.	Results – Quality Assessment paragraph
	20b	Present results of all statistical syntheses conducted. If meta-analysis was done, present for each the summary estimate and its precision (e.g. confidence/credible interval) and measures of statistical heterogeneity. If comparing groups, describe the direction of the effect.	Results – RFS, PFS, AE sections

Section and Topic	Item #	Checklist item	Location where item is reported
	20c	Present results of all investigations of possible causes of heterogeneity among study results.	Results – RFS and PFS sections
	20d	Present results of all sensitivity analyses conducted to assess the robustness of the synthesized results.	Results – Sensitivity analyses paragraph
Reporting biases	21	Present assessments of risk of bias due to missing results (arising from reporting biases) for each synthesis assessed.	Results – Funnel plots (Figure 3)
Certainty of evidence	22	Present assessments of certainty (or confidence) in the body of evidence for each outcome assessed.	Results – Table 2 (GRADE)
**DISCUSSION**			
Discussion	23a	Provide a general interpretation of the results in the context of other evidence.	Discussion, paragraphs 1–3
	23b	Discuss any limitations of the evidence included in the review.	Discussion – Limitations paragraph
	23c	Discuss any limitations of the review processes used.	Discussion – Limitations paragraph
	23d	Discuss implications of the results for practice, policy, and future research.	Discussion – Final paragraphs
**OTHER INFORMATION**			
Registration and protocol	24a	Provide registration information for the review, including register name and registration number, or state that the review was not registered.	Methods – PROSPERO registration
	24b	Indicate where the review protocol can be accessed, or state that a protocol was not prepared.	Methods – PROSPERO registration
	24c	Describe and explain any amendments to information provided at registration or in the protocol.	Not applicable
Support	25	Describe sources of financial or non-financial support for the review, and the role of the funders or sponsors in the review.	Acknowledgments

Section and Topic	Item #	Checklist item	Location where item is reported
Competing interests	26	Declare any competing interests of review authors.	Conflicts of Interest
Availability of data, code and other materials	27	Report which of the following are publicly available and where they can be found: template data collection forms; data extracted from included studies; data used for all analyses; analytic code; any other materials used in the review.	Data Availability Statement
